# Healthcare as a development engine: access, employment, and sustainable growth in Serbia

**DOI:** 10.3389/fpubh.2026.1817268

**Published:** 2026-04-10

**Authors:** Timotej Jagrič, Jovan Zubović, Aleksandar Zdravković, Aljaž Herman

**Affiliations:** 1 Faculty of Economics and Business, Institute of Finance and Artificial Intelligence, University of Maribor, Maribor, Slovenia; 2 Institute of Economic Sciences, Beograd, Serbia

**Keywords:** economic development, economic footprint, government policy, health sector, input–output tables, multipliers

## Abstract

**Background:**

In low- and middle-income countries like Serbia, improving access to healthcare is a central objective of social and development policy, closely linked to the Sustainable Development Goals, particularly SDG 3 (Good Health and Well-Being) and SDG 8 (Decent Work and Economic Growth). However, healthcare expenditure is still frequently perceived as a fiscal cost rather than as a productive investment. Serbia, an middle-income country with a predominantly publicly financed healthcare system, offers a relevant case for examining how expanding healthcare access is intertwined with domestic economic structures and inclusive development outcomes.

**Methods:**

Using national input–output tables for 2019, this study applies standard input–output modelling to estimate output, income, employment, and value-added multipliers associated with the Serbian healthcare sector. The analysis captures direct, indirect, and induced effects, enabling an assessment of how healthcare spending propagates through domestic supply chains and labor markets.

**Results:**

The findings show that healthcare in Serbia generates significant economy-wide spillovers that extend well beyond service provision. Output multipliers exceed unity, indicating strong domestic production linkages that support the availability and affordability of healthcare services. Employment multipliers rank healthcare among the leading job-generating sectors, contributing directly to SDG 8 by fostering stable and locally anchored employment. Most notably, the total value-added multiplier places healthcare among the top ten sectors of the Serbian economy, underscoring its capacity to retain expenditure domestically and to reinforce the economic foundations necessary for sustained healthcare access.

**Conclusion:**

The results demonstrate that investments aimed at improving healthcare access simultaneously advance broader economic inclusion and sustainable development objectives. Comparative benchmarking with European economies reveals structurally consistent patterns in health-sector integration across income levels, while also highlighting country-specific differences in income and employment transmission. By empirically linking healthcare access to macroeconomic returns, this study provides evidence to support policy frameworks that treat health expenditure as a strategic investment for achieving SDG-aligned, resilient, and inclusive growth in low- and middle-income countries.

## 1 Introduction

Advances in medical services, technologies, and treatments, together with broader economic development, have intensified scholarly debate on the relationship between health expenditures and health outcomes. However, this relationship is complex and influenced by multiple factors beyond simple financial investment.

On the one hand, inefficiencies in healthcare markets—arising from asymmetric information, adverse selection, and institutional constraints—can limit the effective translation of spending into improved health outcomes (1–3). Hanefeld et al. (1) emphasize that perceptions of quality, responsiveness, and patient-provider trust shape healthcare utilization, while Spiteri and von Brockdorff (3) highlight the non-linear effects of economic growth on health outcomes, showing that higher income does not automatically reduce mortality rates.

On the other hand, social, demographic, and spatial determinants play a crucial role in shaping health outcomes and access to care (4–11). For example, Kinge et al. (7) show that the economic burden of disease varies by type and population, while Schmittdiel et al. (8) demonstrate improvements in access through policy interventions. Espinosa-González et al. (4) and Jagrič et al. (5) illustrate how the structure and governance of health systems influence outcomes, and studies on rural communities (6, 10) highlight the interplay of local resources, geographic dispersion, and service availability. Teig et al. (11) further emphasize that governance determinants, such as policies and institutional power, can either mitigate or exacerbate health inequities.

As a result, higher health expenditure does not necessarily translate into improved access, equity, wellbeing, or regional and community development outcomes.

To address these complexities, researchers have employed a variety of theoretical perspectives, methodological frameworks, estimation techniques, and data sources to study the economic effects of healthcare financing. Some studies focus on input–output models to quantify the economic impact of healthcare spending. For example, Jewczak and Suchecka (12) demonstrate how input–output models can identify strengths and weaknesses in health system performance at national and regional levels. Similarly, Yamada and Imanaka (13) use input–output analysis to estimate the economic impact of all medical institutions in Japan, showing that medical spending generates two to three times its value in economic returns.

Other studies examine healthcare’s interrelations with broader economic structures. Gutiérrez-Hernández and Abásolo-Alessón (14) analyze the health care sector in the European Union using input–output analysis, revealing its role in value added, employment, and productivity across countries. Jagrič et al. (15) extend this approach to 19 European economies, demonstrating that healthcare spending has consistently positive effects on national income, employment, and household wellbeing, with particularly strong impacts in lower-GDP countries.

These diverse approaches illustrate the multifaceted ways in which healthcare financing interacts with economic outcomes at both local and global levels.

Demand-side approaches often conceptualize health as an element of human capital incorporated into individual utility functions, where optimal health consumption is constrained by income and budgetary trade-offs (16). In contrast, supply-side frameworks model healthcare through production functions to estimate attainable health outcomes based on combinations of production inputs, albeit under strong assumptions regarding technical feasibility and exogeneity (17–19).

Complementing these perspectives, a sectoral input–output approach—adopted in this study—examines the healthcare sector’s interdependencies within the national economy, enabling a comprehensive assessment of both direct and indirect economic effects across sectors (12, 13, 20). Within this framework, healthcare spending is analyzed not only as a determinant of health outcomes but also as a source of output, value added, income, employment, and import effects transmitted through domestic production networks. Recent empirical evidence suggests that these multiplier effects are not development-neutral. Using harmonized input–output data for European Union countries, Jagrič et al. (15) show that healthcare multipliers vary systematically with the level of economic development, reflecting bigger structural differences in production technologies, labor productivity, import propensities, and institutional organization across economies.

These findings highlight the importance of situating country-specific input–output results within an explicit comparative and developmental context. Since gross domestic product per capita serves as a proxy for a wide range of structural characteristics—such as capital intensity, domestic supply capacity, and sectoral specialization—benchmarking healthcare multipliers against cross-country income patterns provides a useful reference for interpreting national results. At the same time, the existing literature cautions that income levels alone cannot fully explain variation in multipliers, which are fundamentally determined by technical coefficients, sectoral value-added shares, and the organization of production and procurement systems.

In Serbia, healthcare services are predominantly publicly funded through compulsory health insurance contributions, ensuring broad access despite a substantial share of out-of-pocket expenditures, which account for 36.5% of total health spending. Public health expenditure as a share of gross domestic product exceeds that of several neighboring European Union countries. Following rapid growth in the early 2000s, public health spending declined from 2013 to 2017, after which it resumed a gradual upward trend, reaching 8.52% of gross domestic product in 2020 (21). Against this backdrop, this study applies a sectoral input–output approach to assess the economic impact of the Serbian healthcare system. The analysis pursues two interrelated objectives. First, it quantifies the output, value-added, income, employment, and import multipliers associated with healthcare demand in Serbia. Second, it benchmarks these multipliers against development-related patterns observed in the European Union, thereby situating Serbia’s healthcare sector within a broader comparative framework and clarifying the extent to which observed effects reflect income-related regularities versus country-specific structural characteristics.

Against this background, this study contributes to the literature in several ways. First, it provides a unique comprehensive input–output assessment of the Serbian healthcare sector using recent national data, offering detailed estimates of output, value-added, income, employment, and import multipliers. Second, by explicitly benchmarking Serbian results against development-related patterns derived from harmonized EU input–output data, the analysis moves beyond standalone multiplier estimation and places national outcomes within a comparative structural framework. This allows us to distinguish between effects driven by income level and those reflecting country-specific production networks, labor-market characteristics, and procurement structures. Third, the results document a clear asymmetry between strong output and employment effects and comparatively weaker income transmission, highlighting the importance of institutional and structural factors in shaping the macroeconomic impact of healthcare spending. Finally, the study provides policy-relevant evidence on the role of healthcare as both a demand stimulus and a development instrument in a middle-income economy, offering empirical insights into ongoing debates over the economic returns to health expenditure and the conditions under which healthcare investment supports sustainable growth.

The remainder of the paper is organized as follows. The next section describes the data, addresses data quality issues, and outlines the testing procedures. This is followed by a presentation of the methodology, empirical results, and a critical discussion of the findings.

## 2 Materials and methods

### 2.1 Data on the Serbian economy and the health sector

Input–output (IO) tables provide a comprehensive representation of the circulation of economic flows within an economy by recording intersectoral transactions in a tabular format. The core of an IO system consists of three quadrants, with the first quadrant capturing intermediate transactions among production sectors (22). Each sector is linked to itself and to all other sectors through individual matrix cells, allowing for the identification of direct and indirect production linkages. Input–output tables can be compiled either with imports integrated into total flows or with imports recorded separately, in which case the system distinguishes between domestic production flows and imported inputs.

Several international and national institutions produce input–output tables that differ in coverage, sectoral detail, and methodological consistency. For Serbia, publicly available sources include: (i) the Statistical Office of the Republic of Serbia (SORS), which provides national input–output tables for 2010, 2015, 2019, and 2020; (ii) Eurostat, which publishes selected tables for 2021; and (iii) the Eora multi-region input–output database, which offers long time-series coverage from 1990 onward. Since SORS is the only provider of primary national input–output data compiled directly from Serbian supply and use statistics, its datasets form the basis of the empirical analysis in this study. While more recent supply and use tables are available from SORS, covering up to 2022, this study relies on symmetric input–output tables, for which more recent versions were not available at the time of the analysis. We acknowledge this as a limitation, as updated input–output tables could potentially alter the results, given that the COVID-19 pandemic significantly affected the structure of the Serbian economy, and subsequent events, such as the Ukraine crisis, may have further influenced intersectoral linkages.

In 2019, SORS compiled and released for the first time a complete and internally consistent set of supply, use, and input–output tables for Serbia for the reference year 2015, following the European System of Accounts (ESA 2010)^1^ and the Eurostat manual for supply, use, and input–output tables.^2^ In accordance with Eurostat’s five-year compilation cycle, a new set of input–output tables was produced for 2020, while the tables for 2010 were subsequently reconstructed using updated national accounts information. As a result, three complete sets of national input–output tables—available in both product-by-product and industry-by-industry formats—are currently publicly accessible for Serbia.

Because the year 2020 was heavily affected by the COVID-19 pandemic and associated disruptions to production, demand, and labor markets, the Institute of Economic Sciences requested the compilation of an additional industry-by-industry input–output table for 2019. This table is considered to better reflect the medium-run structural characteristics of the Serbian economy under non-crisis conditions. The 2019 input–output table is valued at basic prices and covers 21 NACE sections disaggregated into 88 divisions at the two-digit level. The main properties of the selected Serbian input–output tables are summarized in Table 1.

**Table 1:** Properties of the selected Serbian input–output tables.

Year	Number of sectors	Product × product	Industry × industry	Separated import flows	The separated health care sector	Data for calculating deflators is available	Sectoral data for employment is available
2010	65	Yes	Yes	No	Yes	No	Yes
2015	65	Yes	Yes	No	Yes	No	Yes
2019	88	No	Yes	No	Yes	No	Yes
2020	65	Yes	Yes	No	Yes	No	Yes

All IO tables are compiled according to the ESA 2010 Methodology and NACE Rev. 2/CPA 2008 classification method.

Source: Statistical Office of the Republic of Serbia.

National input–output tables possess several features that make them particularly suitable for applied economic research (23). They provide a coherent accounting framework linking production, income generation, and final demand, enable the estimation of sectoral multipliers and inter-industry spillovers, and facilitate cross-country comparisons of structural characteristics. At the same time, their practical use is subject to several limitations. Input–output analysis relies on restrictive assumptions of fixed technical coefficients and linear production relationships, which constrain the interpretation of results, especially in long-term or counterfactual settings (24). Additional challenges include changes in sectoral classifications over time, the need for supplementary aggregation (25), and the absence of price adjustments unless explicit deflation procedures are applied.

The quality of the available Serbian input–output tables is assessed in Tables 2–4 by comparing key aggregates with corresponding figures from the national accounts. Table 2 contrasts the production structure implied by the input–output tables with GDP by activity, Table 3 compares expenditure-side aggregates, and Table 4 evaluates consistency with the income-based decomposition of GDP. This assessment indicates that sufficiently reliable and internally consistent data for multiplier analysis are available only for the year 2019. The 2010 table exhibits substantial discrepancies relative to national accounts aggregates, while the 2015 table lacks information on income components, limiting its applicability for income-multiplier estimation. Moreover, detailed and consistent sectoral employment data are available only for 2019, which is essential for employment multiplier analysis. Finally, the 2020 input–output table is strongly affected by pandemic-related distortions and is therefore unlikely to represent normal economic conditions.

**Table 2:** Comparison of data in input–output tables with GDP production structure.

Year	Aggregate	Total all sectors	IOT	Difference	Net taxes on products
2010	Production	5,826,004	6,896,660	+18.4%	
	Intermediate consumption	3,425,963	4,179,679	+22.0%	
	Value added	2,400,041	2,716,981	+13.2%	587,099
	Gross domestic product	2,881,891	3,304,081	+14.6%	
2019	Production	11,186,390	11,186,402	0.0%	
	Intermediate consumption	6,701,437	6,701,451	0.0%	
	Value added	4,484,953	4,484,951	0.0%	944,273
	Gross domestic product	5,421,851	5,429,224	+0.1%	
2020	Production	11,123,986	11,165,368	+0.4%	
	Intermediate consumption	6,551,929	6,591,098	+0.6%	
	Value added	4,572,056	4,574,269	+0.0%	938,039
	Gross domestic product	5,502,216	5,512,308	+0.2%	

Current prices in mil. RSD.

Source: Statistical Office of the Republic of Serbia (Statistical yearbook).

**Table 3:** Comparison of data in input–output tables with GDP expenditure structure.

Year	Aggregate	Total all sectors	IOT	Difference
2010	Final consumption expenditure of households	2,421,050	2,408,053	−0.5%
	Final consumption expenditure of NPISH	32,524	32,505	−0.1%
	Final consumption expenditure of general government	622,120	622,120	0.0%
	Gross capital formation	574,556	574,489	0.0%
2015	Final consumption expenditure of households	3,052,788	3,048,010	−0.2%
	Final consumption expenditure of NPISH	51,814	51,815	0.0%
	Final consumption expenditure of general government	708,160	708,160	0.0%
	Gross capital formation	732,284	732,284	0.0%
2019	Final consumption expenditure of households	3,636,792	3,615,984	−0.6%
	Final consumption expenditure of NPISH	62,269	62,269	0.0%
	Final consumption expenditure of general government	901,124	901,124	0.0%
	Gross capital formation	1,218,007	1,218,007	0.0%
2020	Final consumption expenditure of households	3,605,824	3,619,055	+0.4%
	Final consumption expenditure of NPISH	60,795	60,795	0.0%
	Final consumption expenditure of general government	962,225	962,225	0.0%
	Gross capital formation	1,180,061	1,180,127	0.0%

Current prices in mil. RSD.

Source: Statistical Office of the Republic of Serbia database (Gross domestic product by expenditure approach).

**Table 4:** Comparison of data in input–output tables with GDP income structure.

Year	Aggregate	Total all sectors	IOT	Difference
2010	Compensation of employees	1,239,051	n/a	
	Gross operating surplus and mixed income	1,307,136	n/a	
2019	Compensation of employees	2,227,378	2,528,257	+13,5%
	Gross operating surplus and mixed income	2,205,576	n/a	
2020	Compensation of employees	2,432,328	n/a	
	Gross operating surplus and mixed income	2,223,234	n/a	

Current prices in mil. RSD.

Source: Statistical Office of the Republic of Serbia (Statistical yearbook).

Based on the 2019 input–output table, Table 5 presents the structure of intermediate inputs used by the health sector. The first three rows report inputs originating from agriculture, industry, and services. Within industrial inputs, pharmaceutical products represent the dominant component, reflecting the central role of medical supplies in healthcare provision. Service-sector inputs are also substantial, with wholesale and business services accounting for the largest shares. This input composition highlights the health sector’s extensive backward linkages to domestic suppliers, which are a key determinant of its multiplier effects.

**Table 5:** Input–output structure of the health sector in 2019, current basic prices.

Sector/industry	mil RSD	mil EUR
*Agriculture*	*1,111*	*9*
*Industry*	*59,062*	*503*
*Services*	*29,355*	*250*
Total intermediate consumption	89,528	762
Total intermediate consumption adjusted for taxes less subsidies	104,639	891
*Value added, gross*	*172,241*	*1,466*
Total output	276,880	2,356
*Import*	*903*	*8*
Total supply	277,783	2,364

Source: Statistical Office of the Republic of Serbia and authors’ calculations. The italic values represent subcategories of total values.

To ensure comparability with existing input–output studies for European Union countries, particularly those examining the relationship between healthcare multipliers and economic development, the original 88-sector classification of the 2019 Serbian input–output table was aggregated to 62 sectors, following the approach proposed by Jagrič et al. (26). This harmonization facilitates subsequent benchmarking of Serbian healthcare multipliers against development-related patterns observed in EU input–output data.

### 2.2 Methods

The methodological framework applied in this study, including notation and multiplier definitions, follows the standard input–output (IO) analysis as presented in Miller and Blair (27). Input–output analysis is based on two fundamental macroeconomic accounting identities. From the output perspective, total sectoral output equals the sum of intersectoral sales and exogenous final demand, which can be expressed as:


x=Z·i+f,

where x denotes the vector of total output across n sectors, Z is the matrix of intersectoral transactions, i is a summation vector, and f represents final demand. From the input perspective, total sectoral output equals the sum of intermediate inputs and value added:

$$x=Z^{T}\cdot i+v,$$

where v denotes the vector of gross value added. In a balanced accounting framework, total output equals total input, implying equivalence of the two identities. These relationships are illustrated using a stylized industry-by-industry input–output table for Serbia for 2019 (Table 6), which aggregates economic activity into three broad sectors: agriculture, industry, and services.

**Table 6:** Stylized 2019 industry × industry I-O table, 2019 basic current prices (mil RSD).

Sector/industry	Agriculture	Industry	Services	Total consumption expenditure	Gross Capital Formation	Exports	Total use
Agriculture	102,888	246,073	97,807	242,232	32,139	133,974	855,114
Industry	179,468	2,437,820	985,264	1,146,579	973,798	1,719,060	7,441,989
Services	111,551	776,996	1,451,610	2,665,379	275,732	767,079	6,048,347
Total intermediate consumption	393,907	3,460,890	2,534,681				
Total intermediate consumption adjusted for taxes less subsidies	424,347	3,572,580	2,704,524				
Value added	322,843	1,387,890	2,774,218				
Total output	747,190	4,960,470	5,478,742				
Import	107,924	2,481,520	569,605				
Total supply	855,114	7,441,989	6,048,347				

Source: Statistical Office of the Republic of Serbia and authors’ calculations.

The core analytical tool of the IO framework is the matrix of technical coefficients A , whose elements are defined as $a_{\mathrm{ij}}=z_{\mathrm{ij}}/x_{j}$ , representing the input from sector i required to produce one unit of output in sector j . The Leontief demand model can then be written as:


x=A·x+f.

Solving for total output yields:


$$x={\left(I-A\right)}^{-1}\cdot f,$$

where ${\left(I-A\right)}^{-1}$ is the Leontief inverse matrix. An exogenous increase in final demand for the output of a given sector therefore generates not only a direct increase in that sector’s production, but also indirect output effects across all supplying sectors. These effects propagate through successive rounds of intersectoral transactions, and their cumulative magnitude defines the indirect component of the multiplier effect.

Following established applications of IO analysis to the healthcare sector (15, 20, 26), sectoral output multipliers are obtained by summing the elements of the relevant column of the Leontief inverse. Table 7 reports the ten industries most directly affected by changes in health-sector output. Together, these industries account for nearly 80% of the total direct production effect associated with an increase in final demand for health services, highlighting the health sector’s strong backward linkages to domestic suppliers.

**Table 7:** Intermediate inputs used by the health sector (10 industries with the highest direct effects).

Industry	Input value (mill RSD)	Direct effect	Contribution (%)
Basic pharmaceutical products and pharmaceutical preparations	18,270	0.0658	20.407
Wholesale trade services, except of motor vehicles and motorcycles	15,637	0.0563	17.466
Furniture and other manufactured goods	12,450	0.0448	13.906
Electricity, gas, steam and air conditioning	6,014	0.0216	6.717
Chemicals and chemical products	4,598	0.0166	5.136
Coke and refined petroleum products	4,448	0.0160	4.968
Human health services	3,084	0.0111	3.445
Food, beverages and tobacco products	2,256	0.0081	2.520
Constructions and construction works	2,067	0.0074	2.309
Rubber and plastic products	2,048	0.0074	2.288
Total	70,872	0.2551	79.162

Source: Statistical Office of the Republic of Serbia and authors’ calculations.

A key modelling choice in multiplier analysis concerns the classification of sectors as endogenous or exogenous. In the basic IO framework, production sectors are treated as endogenous, while final demand components are treated as exogenous. Under this assumption, an exogenous increase in final demand affects output through inter-industry linkages, but does not generate feedback effects on final demand itself. This simplification is particularly restrictive for households that both earn income from production and spend that income on consumption.

**Figure 1:**
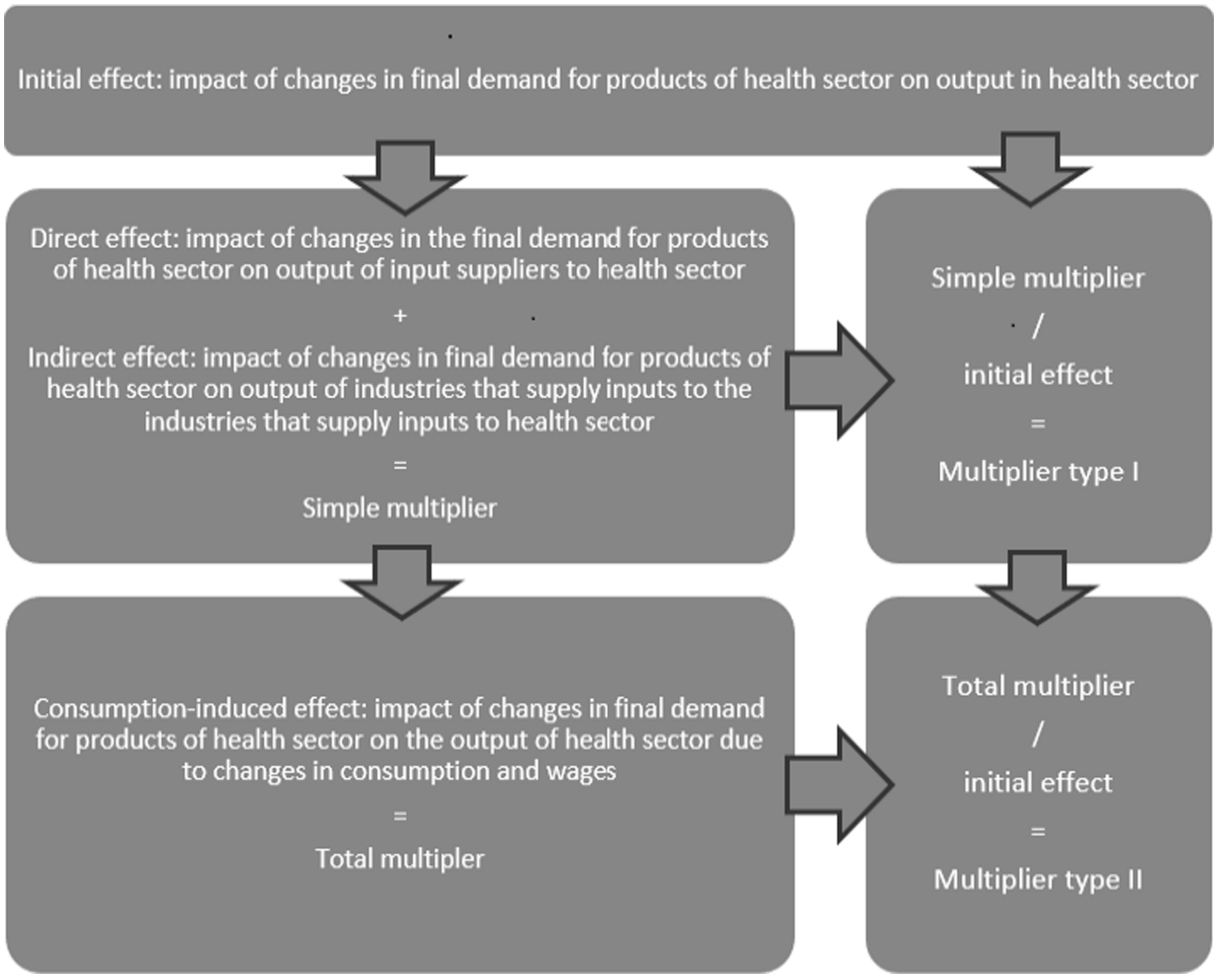
Types of multipliers. Source: Authors.

To account for income–consumption feedback effects, the household sector can be endogenized by removing household consumption from final demand and employee compensation from gross value added, and including them instead as an additional column and row in the technical coefficient matrix (27). This extension allows for the computation of different types of multipliers. When households are treated as exogenous, the resulting multipliers capture only direct and indirect effects and are commonly referred to as simple multipliers. When households are endogenized, induced effects arising from household income and consumption are incorporated, yielding total multipliers. In addition, type I and type II multipliers can be defined to measure the propagation of sector-specific changes through the economy. The relationships among simple, total, type I, and type II multipliers for the health sector are illustrated in Figure 1 (27, 28).

The empirical analysis focuses on simple and total multipliers, as simple multipliers provide a natural basis for cross-country benchmarking and comparison with harmonized European Union input–output studies, while total multipliers capture the full income–consumption feedback effects within the Serbian economy. To compute total multipliers, the household sector must be endogenized, which requires additional information on employee compensation and household disposable income.

Although Serbian input–output tables report gross value added, they do not yet provide a full decomposition of value added into compensation of employees, consumption of fixed capital, and operating surplus for all sectors. To address this limitation, sectoral compensation of employees was estimated by applying labor cost shares derived from the annual Structural Business Survey^3^ (SBS) to the corresponding sectoral value-added figures in the input–output table. The SBS covers the non-financial business sector^4^ and provides consistent information on labor costs relative to value added. For the financial sector, compensation of employees was approximated using official statistics on annual gross wages.

Data on household gross disposable income are not published in a form directly compatible with the input–output accounting framework. While the Statistical Office of the Republic of Serbia (SORS) reports household income information through the Household Budget Survey^5^ (HBS), these data are not directly valued at current basic prices. To approximate household gross disposable income, the total compensation of employees obtained from the input–output table was multiplied by the ratio of total household income to household income from regular wages and salaries as reported by the HBS. For 2019, this ratio equals 2.03. This procedure endogenizes household consumption in a manner consistent with the structure of the Serbian input–output table, enabling the computation of total multipliers for the health sector.

## 3 Results

### 3.1 Output and income multipliers

We first consider the estimated output multipliers for the Serbian health sector. Table 8 reports simple, total, type I, and type II output multipliers for 2019, alongside the corresponding national averages. For benchmarking purposes, the table also includes the estimated multipliers for the Slovenian health sector reported in Bekő et al. (28). The total output multiplier indicates that a €1 million increase in final demand for health services generates a total increase in gross output of approximately €2.8 million across the Serbian economy. In relative terms, the health sector ranks 32nd out of 62 sectors, implying that changes in final demand in 31 sectors generate a larger total output response.

**Table 8:** Output multipliers for Serbian health sector (current prices, 62 sectors).

Type of multiplier	Set	Serbia 2019			Slovenia 2014	
		Value	Average	Rank	Value	Rank
Output multiplier	Simple	1.5609	1.7769	46	1.2924	44
	Total	2.7793	2.6733	32	2.8317	17
	Truncated	2.2034	2.2496	38	2.0298	32

Value: level of the Human health services multiplier; Average: simple average of level of multipliers for all sectors in the economy; Rank: rank of Human health services multiplier in comparison to multipliers of other sectors in the economy.

Source: Statistical Office of the Republic of Serbia and authors’ calculations.

Compared with available estimates for Slovenia, total output multipliers for the health sector are of similar magnitude, although the sector’s relative position differs. In Slovenia, the health sector ranked 17th out of 49 sectors, suggesting a stronger relative propagation of health-sector demand through inter-industry production linkages. These differences point to variation in the structure and length of domestic supply chains associated with health services across countries.

**Figure 2:**
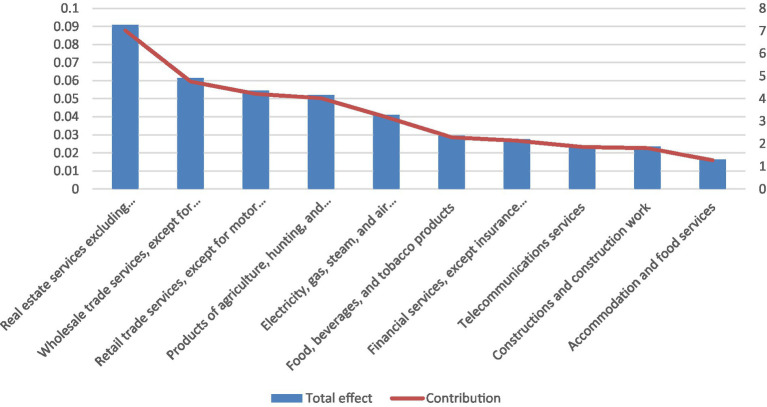
Top 10 industries (apart from the health sector) whose output is affected by changes in the final demand for health services, based on the total multiplier. Source: Statistical Office of the Republic of Serbia and authors’ calculations.

Figure 2 lists the ten industries—excluding the health sector itself—most affected by changes in final demand for health services, based on total output multipliers. The wholesale trade sector emerges as the most sensitive, with a €1 million increase in health-sector demand generating approximately €120,000 in additional wholesale output, corresponding to about 4.2% of total output multiplication. Other strongly affected sectors include manufacturing industries supplying pharmaceuticals and medical products, utilities, and selected business services, highlighting the health sector’s reliance on a broad set of domestic intermediate inputs.

To place the Serbian results in a broader comparative context, simple output multipliers are benchmarked against development-related patterns observed in European Union input–output data. Using the estimated EU-2010 cross-sectional relationship between simple output multipliers and GDP per capita, the benchmark predicts a simple output multiplier of approximately 1.46 for an economy at Serbia’s 2019 income level expressed in 2010 euros. The empirically estimated simple output multiplier for Serbia equals 1.56, exceeding the benchmark prediction by about 6.7%. This indicates that, conditional on income level, the Serbian health sector exhibits relatively strong backward production linkages compared with the EU-2010 cross-sectional average.

We next turn to income multipliers, which measure the response of employee compensation to changes in final demand. Income multipliers are computed using a vector of income coefficients defined as the ratio of compensation of employees to total sectoral output. Table 9 reports the estimated income multipliers for the Serbian health sector in 2019, together with the national average and the corresponding estimates for Slovenia.

**Table 9:** Income multipliers for Serbian health sector (current prices, 62 sectors).

Type of multiplier	Set	Serbia 2019			Slovenia 2014	
		Value	Average	Rank	Value	Rank
Income multiplier	Simple	0.4595	0.3381	13	0.586651	4
	Total	0.5759	0.4237	13	0.762999	4
	Type I	1.2253	2.0923	57	1.182212	47
	Type II	1.5356	2.6222	57	1.537587	47

Value: level of the Human health services multiplier; Average: simple average of level of multipliers for all sectors in the economy; Rank: rank of Human health services multiplier in comparison to multipliers of other sectors in the economy.

Source: Statistical Office of the Republic of Serbia and authors’ calculations.

The total income multiplier indicates that a €1 million increase in final demand for health services generates approximately €0.58 million in additional household income. In relative terms, the health sector ranks 13th out of 62 sectors, meaning that only 12 sectors generate a larger total income effect. This places healthcare among the sectors with the strongest income transmission effects in the Serbian economy. In comparison, the Slovenian health sector exhibits a higher total income multiplier and ranks 4th out of 49 sectors, suggesting a relatively stronger wage-income response.

Type I and type II income multipliers are considerably lower ranked in both countries (57th out of 62 sectors in Serbia and 47th out of 49 sectors in Slovenia for type II multipliers). This pattern indicates that while increases in final demand for health services generate substantial income effects, an exogenous increase in income originating within the health sector itself does not induce strong secondary income effects in other sectors. In other words, income propagation is primarily driven by demand-side shocks rather than by income feedback originating within the sector.

Benchmarking against the EU-2010 income multiplier relationship yields a predicted simple income multiplier of approximately 0.54 for Serbia’s income level, while the empirically estimated value equals 0.46. The benchmark, therefore, overpredicts the realized income effect by roughly 18%. This gap suggests that, relative to the EU cross-sectional pattern, health-sector demand in Serbia is associated with a smaller expansion of wage income, potentially reflecting lower labor shares, differences in wage-setting institutions, or the composition of upstream sectors affected by health-sector demand.

**Figure 3:**
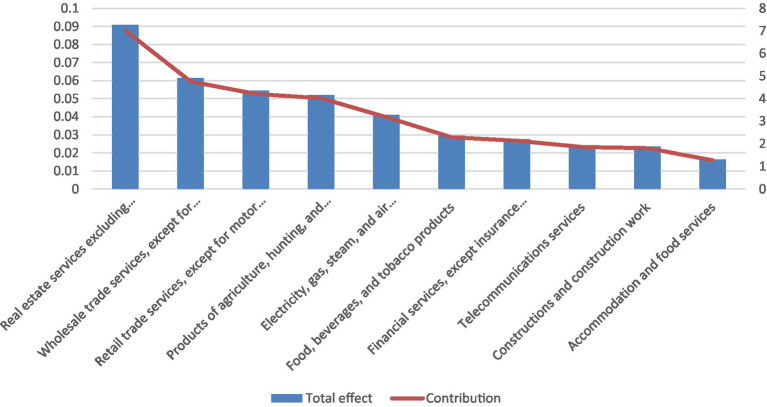
Top 10 industries (apart from the health sector) whose income is affected by changes in the final demand for health services, based on the total multiplier. Source: Statistical Office of the Republic of Serbia and authors’ calculations.

Finally, Figure 3 reports the ten industries—excluding the health sector—whose income is most affected by changes in final demand for health services, based on total income multipliers. As in the case of output effects, the wholesale trade sector plays a prominent role, accounting for approximately 3.8% of total income multiplication. This overlap between output and income effects underscores the central position of wholesale and distribution services in transmitting health-sector demand through the Serbian economy.

### 3.2 Employment multiplication

Following the calculation of income multipliers, we next examine employment multipliers, which measure the effects of changes in final demand on the physical number of employed persons across sectors. The simple employment multiplier captures the total number of jobs generated by an exogenous increase in final demand, excluding consumption-induced effects, while the total employment multiplier additionally incorporates induced employment effects arising from household income and consumption feedbacks.

The computation of employment multipliers requires detailed sectoral employment data. In Serbia, employment statistics are available from two main sources: registered employment statistics^6^ and the Labor Force Survey^7^ (LFS).

While the LFS provides broader coverage, including informal employment, it does not offer sufficient sectoral disaggregation at the two-digit NACE level required for input–output analysis. Consequently, registered employment data are used for most sectors. An exception is agriculture, where LFS-based employment figures are applied due to the large share of informal employment and the resulting discrepancy between registered and actual employment levels.

Table 10 reports the estimated employment multipliers for the Serbian health sector in 2019, alongside the national average and the corresponding estimates for Slovenia reported in Bekő et al. (28). The total employment multiplier indicates that an increase in final demand for health services of RSD 1 billion (approximately €8.52 million) generates 644 additional jobs in the Serbian economy. This corresponds to roughly 76 new jobs per €1 million of increased final demand. In relative terms, the health sector ranks 10th out of 62 sectors, implying that only nine sectors generate a larger total employment effect.

**Table 10:** Employment multipliers for the Serbian health sector (current prices, 62 sectors).

Type of multiplier	Set	Serbia 2019			Slovenia 2014	
		Value	Average	Rank	Value	Rank
Employment multiplier	Simple	0.5166	0.3089	10	2.19E−05	12
	Total	0.6445	0.3853	10	3.04E−05	11
	Type I	1.1670	2.2438	59	1.191253	47
	Type II	1.4557	2.7988	59	1.655021	41

Value: level of the Human health services multiplier; Average: simple average of level of multipliers for all sectors in the economy; Rank: rank of Human health services multiplier in comparison to multipliers of other sectors in the economy.

Source: Statistical Office of the Republic of Serbia and authors’ calculations.

Compared with the Slovenian estimates, the Serbian total employment multiplier appears larger in absolute terms. However, this difference primarily reflects the use of different monetary units and price levels (RSD versus EUR) in the construction of employment coefficients. When employment effects are expressed relative to final demand, the relative ranking of the health sector remains broadly comparable across the two countries.

After normalizing simple and total employment multipliers by the initial direct employment effect, type I and type II employment multipliers for Serbia and Slovenia are broadly similar. In both countries, the health sector ranks substantially lower according to type II multipliers than according to simple and total multipliers (59th out of 62 sectors in Serbia and 41st out of 49 sectors in Slovenia). This pattern mirrors the results for income multipliers and indicates that an exogenous increase in employment in the health sector does not generate strong secondary employment effects in other sectors. In contrast, changes in final demand for health services produce relatively pronounced employment effects through inter-industry linkages.

**Figure 4:**
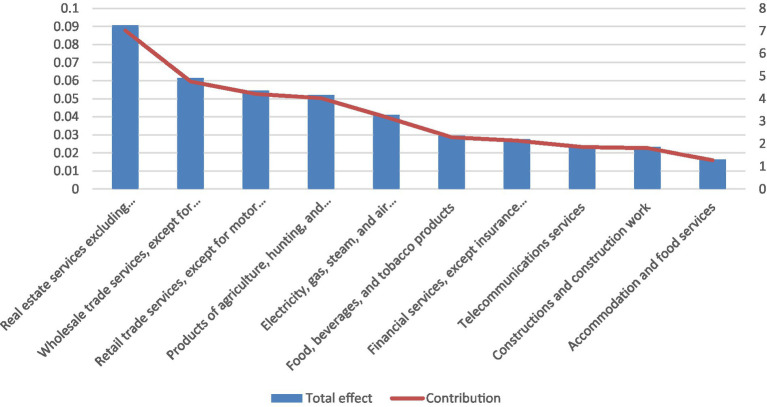
Top 10 industries (apart from the health sector) whose employment is affected by changes in the final demand for health services, based on the total multiplier. Source: Statistical Office of the Republic of Serbia and authors’ calculations.

Finally, Figure 4 presents the ten industries—excluding the health sector—that are most affected by changes in final demand for health services, based on total employment multipliers. Three sectors—agriculture, retail trade, and wholesale trade—also appear among the sectors most affected by income multipliers, although their relative rankings differ. In the case of employment, agriculture and retail trade are particularly sensitive, accounting for approximately 4.9 and 3.8% of total employment multiplication, respectively. This reflects the relatively high labor intensity of these sectors and their role in absorbing indirect employment effects generated by health-sector demand.

### 3.3 Value-added multiplication

Gross value added (GVA) comprises three main components: compensation of employees, consumption of fixed capital (depreciation), and gross operating surplus. Value-added multipliers are computed analogously to income multipliers, using a vector of value-added coefficients defined as the ratio of sectoral gross value added to total output.

Table 11 reports the estimated value-added multipliers for the Serbian health sector in 2019, together with the corresponding national averages and the Slovenian estimates reported in Bekő *et al.* (28). The total value-added multiplier indicates that a €1 million increase in final demand for health services generates approximately €1.3 million in additional gross value added at the national level. In relative terms, the health sector ranks 9th out of 62 sectors, implying that only eight sectors generate a larger total value-added response.

**Table 11:** Value-added multipliers for the Serbian health sector (current prices, 62 sectors).

Type of multiplier	Set	Serbia 2019			Slovenia 2014	
		Value	Average	Rank	Value	Rank
Value-added multiplier	Simple	0.7749	0.5590	9	0.7555	20
	Total	1.2920	0.9320	9	1.1296	12
	Type I	1.2498	2.0074	57	1.2390	43
	Type II	2.0838	3.3470	57	1.8522	40

Value: level of the Human health services multiplier; Average: simple average of level of multipliers for all sectors in the economy; Rank: rank of Human health services multiplier in comparison to multipliers of other sectors in the economy.

Source: Statistical Office of the Republic of Serbia and authors’ calculations.

Compared with the Slovenian estimates, total value-added multipliers for the health sector are broadly similar in magnitude and relative position. In Slovenia, the health sector ranked 12th out of 49 sectors, indicating comparable capacity to translate health-sector demand into domestic value creation. This similarity contrasts with the somewhat larger cross-country variation observed for output and income multipliers.

After normalizing simple and total value-added multipliers by the initial direct value-added effect, type I and type II multipliers for Serbia and Slovenia are also similar in magnitude. As in the case of income and employment multipliers, the relative ranking of the health sector declines substantially when measured by type II multipliers (57th out of 62 sectors in Serbia and 40th out of 49 sectors in Slovenia). This pattern indicates that while final-demand shocks to health services generate strong value-added effects, value-added originating within the health sector itself does not induce pronounced secondary value-added effects in other sectors.

**Figure 5:**
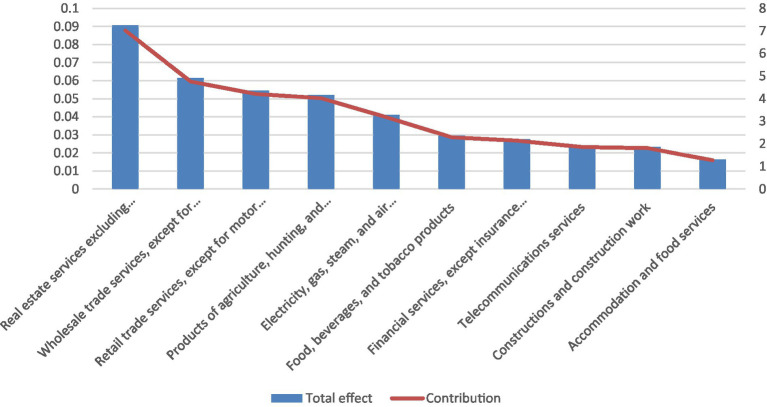
Top 10 industries (apart from the health sector) whose value-added is affected by changes in the final demand for health services, based on the total multiplier. Source: Statistical Office of the Republic of Serbia and authors’ calculations.

Finally, Figure 5 reports the ten industries—excluding the health sector—whose value added is most affected by changes in final demand for health services, based on total value-added multipliers. The real estate services sector emerges as the most sensitive, accounting for approximately 7% of total value-added multiplication. Wholesale trade, retail trade, and agriculture also exhibit high sensitivity, mirroring the patterns observed for income and employment multipliers. This consistency across multiplier types underscores the central role of the service and distribution sectors in transmitting health-sector demand through domestic value-creation channels.

### 3.4 Relationship between simple multipliers and GDP per capita

**Figure 6:**
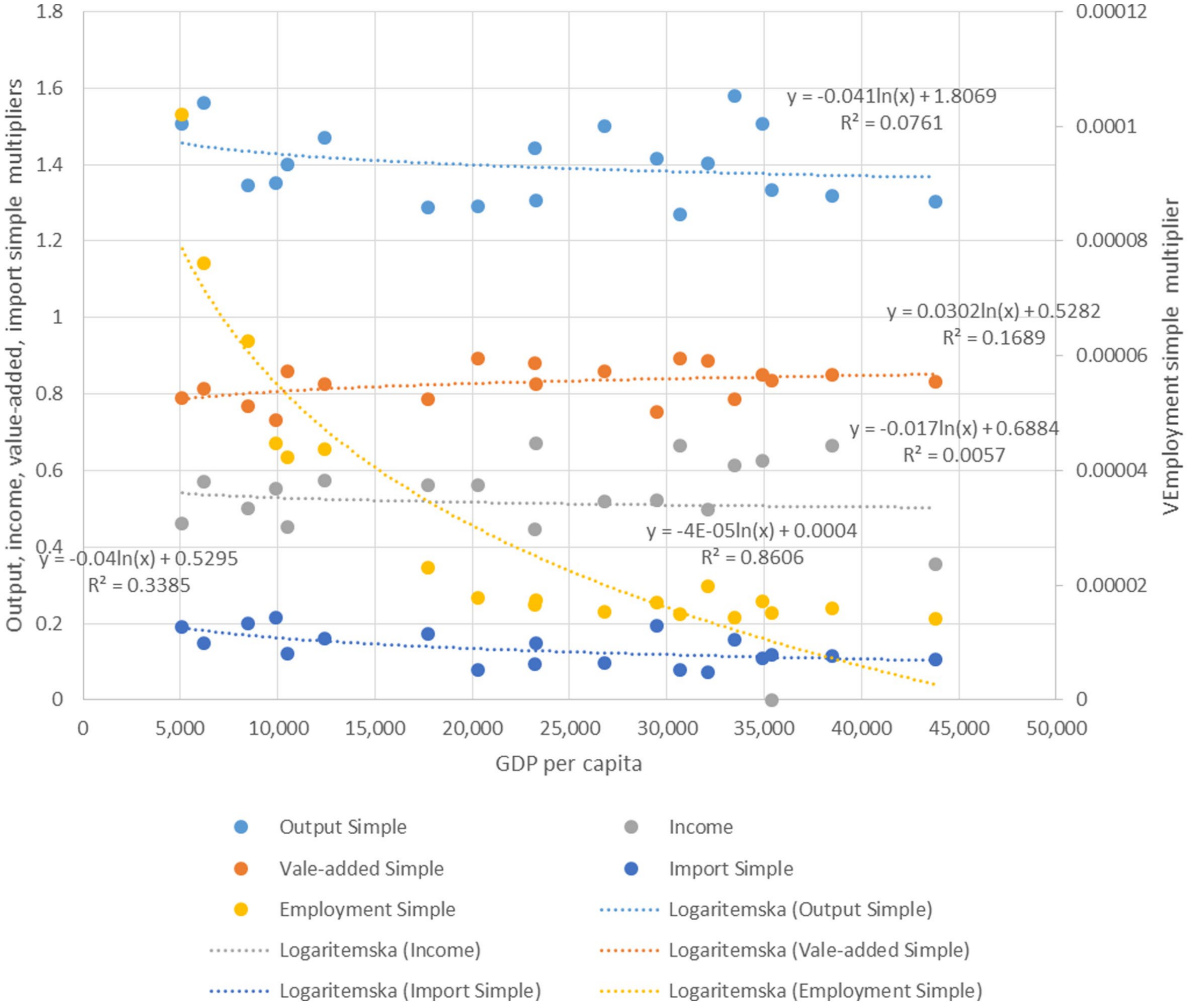
Relationship between simple multipliers and GDP per capita. Source: Author won calculations based on Jagrič et al. (15) study.

To place the Serbian results in a broader development-related context, we benchmark all estimated simple health-sector multipliers—output, value added, income, and employment—against the EU-2010 cross-sectional relationships with GDP per capita (Figure 6).

Results for 2019 indicate that the Serbian health sector exhibits simple multipliers of 1.5609 for output, 0.4595 for income, 0.7749 for value added, and an employment simple multiplier of 0.5166 (unit-dependent). For comparability with the EU-2010 curves, Serbia’s nominal GDP per capita in 2019 (EUR 6,927.7) is deflated by the CPI, yielding an income level of approximately EUR 5,030 in 2010 prices, which is used as input into the benchmark models.

At this income level, the EU-2010 relationships predict a simple output multiplier of 1.457, a value-added multiplier of about 0.79, an income multiplier of 0.543, and a simple employment multiplier of 5.85 × 10^−5^ jobs per euro of final demand (approximately 59 jobs per €1 million). Comparing these benchmarks with Serbia’s observed multipliers yields several insights.

First, Serbia’s realized simple output multiplier exceeds the benchmark by roughly 6.7%, indicating stronger backward production linkages of health services to domestic suppliers than the EU-2010 income-level pattern implies.

Second, the simple value-added multiplier (0.7749) is extremely close to the benchmark prediction, with a deviation of only about −1.4%, suggesting that—conditional on income level—the translation of health-sector demand into domestic gross value added in Serbia closely follows the EU-2010 cross-sectional regularity.

Third, the benchmark income multiplier exceeds the empirical estimate by around 18%. This gap likely reflects structural differences in labor shares, wage pass-through mechanisms, and the sectoral composition of indirect effects, which tend to influence income multipliers more strongly than value-added multipliers.

Finally, the benchmark implies a simple employment multiplier of roughly 59 jobs per €1 million of final demand. Since our empirical results are reported in terms of total employment multipliers (approximately 76 jobs per €1 million), we adjust this figure using the simple-to-total employment multiplier ratio from Table 10 (0.5166/0.6445 ≈ 0.80), yielding an implied simple employment multiplier of about 61 jobs per €1 million. This is very close to the benchmark prediction, with a deviation of approximately −4%, indicating that Serbia’s health-sector employment multiplier is broadly consistent with what would be expected at its income level based on the EU-2010 cross-section.

Overall, EU-2010 benchmarking suggests that Serbia’s 2019 health-sector multipliers—particularly for value added and employment—are closely aligned with development-related patterns observed across EU economies once GDP per capita is expressed in comparable (2010) prices. At the same time, Serbia exhibits somewhat stronger output multipliers and weaker income multipliers than predicted. Given the low explanatory power of GDP per capita for output and income multipliers in the EU cross-section, these deviations are best interpreted as reflecting meaningful structural differences in production networks, import leakages, wage structures, and procurement practices rather than model error. GDP per capita thus serves only as a proxy for deeper technical and institutional characteristics of the health-care value chain.

This interpretation is consistent with health-economics theory, which emphasizes that rising income increases demand for health services while production remains labor-intensive, without implying a unique mapping from income to input–output multipliers (16). Accordingly, the Serbian comparison should be viewed as a benchmarking exercise rather than evidence of a causal development effect.

## 4 Conclusion and policy implications

An analysis of 2019 input–output tables shows that the healthcare sector in Serbia is closely linked to the domestic economy and exerts significant effects through cross-sectoral linkages. Increased demand for health services activates a wide range of suppliers, mainly in service activities such as wholesale and retail trade, municipal and business services, and to a lesser extent in agriculture. This reflects the structure of intermediate inputs in healthcare and the consumption patterns of households that receive income from healthcare activities.

Production multipliers are well above 1, and total multipliers (including household consumption effects) exceed 2, comparable to results for Slovenia and other European countries. This shows that healthcare spending in Serbia triggers a large expansion of economic activity outside the sector itself and that the feedback links with domestic suppliers are relatively strong, while at the same time limiting immediate leakage to imports.

The effects on employment are also significant. An increase in final demand of RSD 1 billion creates approximately 644 new jobs across the economy, placing the health sector among the ten activities with the largest total employment effect. However, Type II multipliers, which measure additional rounds of employment relative to the initial effect, are relatively low. This means that most new jobs are created directly in healthcare and its direct suppliers, while further effects through increased household consumption are limited.

A similar pattern is seen in value added. Healthcare ranks high in terms of its overall impact on national value added, reflecting strong links to domestic production and relatively low dependence on imports. At the same time, lower results for type I and II multipliers indicate that the value added generated in healthcare is transferred only to a limited extent to additional value-creation cycles in other activities.

Income multipliers also confirm that increased demand for healthcare services does increase total household income, but this income does not flow strongly into further cycles of cross-sectoral growth. The reasons are likely to be related to the wage structure, the share of labor in individual industries, and the composition of healthcare-related supply chains.

A comparison with European reference patterns (EU-2010) shows that Serbian value-added and employment multipliers are roughly in line with what would be expected given the level of income, while output multipliers are slightly higher and income multipliers are lower than expected. This highlights that GDP per capita explains only a small part of the differences between countries; the national structure of production, import dependence, and the institutional characteristics of the labor market and public procurement play a decisive role.

From an economic policy perspective, the results show that health spending in Serbia has a relatively strong impact on domestic production and employment and can therefore serve as an effective short-term demand stabilizer, especially during periods of economic weakness. Compared to more capital-intensive investments, health spending activates domestic supply chains more quickly and creates jobs.

At the same time, the difference between strong production effects and weaker income effects suggests that increased health spending does not, in itself, guarantee broad-based income growth. Accompanying measures are key to this: improving productivity, developing the skills and competencies of the healthcare workforce, strengthening domestic production of medical inputs, and more thoughtful procurement practices.

At the same time, the results caution that transferring multipliers from other countries without accounting for the domestic economic structure can lead to misleading estimates. National input–output linkages remain a key framework for understanding actual macroeconomic effects.

Overall, the analysis shows that healthcare in Serbia is an important driver of output and employment, but its long-term effects on income and productivity depend heavily on structural reforms. It therefore makes sense to view healthcare spending not only as a social policy but also as part of a broader development strategy, the effectiveness of which varies with a country’s development status and the organization of its economy.

## Data Availability

The raw data supporting the conclusions of this article will be made available by the authors, without undue reservation.

## Footnotes

1
https://ec.europa.eu/eurostat/web/products-manuals-and-guidelines/-/ks-02-13-269

2
https://www.stat.gov.rs/en-US/oblasti/nacionalni-racuni/godisnji-nacionalni-racuni-ponuda-i-upotreba

3
https://data.stat.gov.rs/Home/Result/190101?languageCode=en-US

4 It is important to mention that SBS data on the agriculture sector (NACE code A) and the services sectors, including health care (NACE codes from P to S), are compiled by the SORS, but not publicly available. At the request of the IES, the SORS has provided the data for 2019.

5
https://publikacije.stat.gov.rs/G2020/Pdf/G20205662.pdf

6
https://data.stat.gov.rs/Home/Result/240203?languageCode=en-US

7
https://data.stat.gov.rs/Home/Result/240002090202?languageCode=en-US
